# Hematopoietic neoplasms in Prkar2a-deficient mice

**DOI:** 10.1186/s13046-015-0257-z

**Published:** 2015-11-25

**Authors:** Emmanouil Saloustros, Paraskevi Salpea, Chen-Feng Qi, Lina A. Gugliotti, Kitman Tsang, Sisi Liu, Matthew F. Starost, Herbert C. Morse, Constantine A. Stratakis

**Affiliations:** Section on Endocrinology and Genetics, Program on Developmental Endocrinology & Genetics (PDEGEN) & Pediatric Endocrinology Inter-institute Training Program, Eunice Kennedy Shriver National Institute of Child Health & Human Development (NICHD), National Institutes of Health (NIH), Bethesda, MD 20892 USA; Laboratory of Immunogenetics, National Institute of Allergy and Infectious Diseases, National Institutes of Health, 5640 Fishers Lane, Rockville, MD 20852 USA; Program in Genomics and Differentiation, Eunice Kennedy Shriver National Institute of Child Health & Human Development (NICHD), National Institutes of Health (NIH), Bethesda, MD 20892 USA; Division of Veterinary Resources, Office of the Director (OD), National Institutes of Health, Bethesda, MD 20892 USA

**Keywords:** Protein kinase A, Histiocytic sarcoma, Cyclic AMP, Diffuse large B cell lymphoma, Carney complex

## Abstract

**Background:**

Protein kinase A (PKA) is a holoenzyme that consists of a dimer of regulatory subunits and two inactive catalytic subunits that bind to the regulatory subunit dimer. Four regulatory subunits (RIα, RIβ, RIIα, RIIβ) and four catalytic subunits (Cα, Cβ, Cγ, Prkx) have been described in the human and mouse genomes. Previous studies showed that complete inactivation of the *Prkar1a* subunit (coding for RIα) in the germline leads to embryonic lethality, while *Prkar1a*–deficient mice are viable and develop schwannomas, thyroid, and bone neoplasms, and rarely lymphomas and sarcomas. Mice with inactivation of the *Prkar2a* and *Prkar2b* genes (coding for RIIα and RIIβ, respectively) are also viable but have not been studied for their susceptibility to any tumors.

**Methods:**

Cohorts of *Prkar1a*^*+/−*^*, Prkar2a*^*+/−*^*, Prkar2a*^*−/−*^, *Prkar2b*^*+/−*^ and wild type (WT) mice have been observed between 5 and 25 months of age for the development of hematologic malignancies. Tissues were studied by immunohistochemistry; tumor-specific markers were also used as indicated. Cell sorting and protein studies were also performed.

**Results:**

Both *Prkar2a*^*−/−*^ and *Prkar2a*^*+/−*^ mice frequently developed hematopoietic neoplasms dominated by histiocytic sarcomas (HS) with rare diffuse large B cell lymphomas (DLBCL). Southern blot analysis confirmed that the tumors diagnosed histologically as DLBCL were clonal B cell neoplasms. Mice with other genotypes did not develop a significant number of similar neoplasms.

**Conclusions:**

*Prkar2a* deficiency predisposes to hematopoietic malignancies in vivo. RIIα’s likely association with HS and DLBCL was hitherto unrecognized and may lead to better understanding of these rare neoplasms.

## Background

Protein Kinase A (PKA) is the main mediator of cyclic adenosine mono-phosphate (cAMP)-dependent signaling [1, 2]. In humans and rodents, PKA is a heterotetramer formed by two regulatory (RIα, RIβ, RIIα, RIIβ) and two catalytic subunits (Cα, Cβ, Cγ, Cx) [1, 3]. The PKA subunits have distinct expression patterns: α-subunits are expressed ubiquitously, whereas β-subunits are expressed in a more tissue-specific fashion. There are two types of PKA holoenzymes, type I and type II, depending on the identity of the regulatory subunit [4]. The cAMP binds to the PKA regulatory subunits (two cAMP molecules per subunit), which leads to dissolution of the tetramer, allowing for the catalytic subunits to act as serine-threonine kinases, phosphorylating numerous targets [1, 2].

Among the regulatory subunits, the PKA regulatory subunit type 1A (PRKAR1A or RIα) has the highest affinity for cAMP and is therefore more sensitive to cAMP, which makes RIα essential for maintaining regulated PKA activity [5]. The *PRKAR1A* gene on human chromosome 17q22-24 is the gene encoding for RIα; mutations in this gene are responsible for the multiple tumor syndrome Carney complex (CNC, Online Mendelian Inheritance in Man #160980) [6, 7]. CNC patients develop myxomas, skin lesions, schwannomas, bone and endocrine tumors, and a variety of cancers but they are not known to be predisposed to hematologic malignancies. Mice haploinsufficient for a null allele of *Prkar1a* (*Prkar1a*^*+/−*^) developed tumors, mainly bone tail and thyroid tumors and schwannomas, but the spectrum was somewhat different from that seen in humans [8]. Mice bearing a transgene expressing an anti-sense *Prkar1a* construct (AS-*Prkar1a*) developed more tumors than the *Prkar1a*^*+/−*^ mice, including adrenal hyperplasia, histiocytic sarcomas (HS) and lymphomas [9]. Taken together, these studies clearly identified *PRKAR1A* as a tumor suppressor gene affecting several cell types in both humans and mice but left it unclear as to whether PKA defects might lead to a predisposition to hematologic malignancies.

Previous studies showed that *Prkar2a* knockout (KO) mice are viable, but a predisposition to tumor development has not been reported [10–12]. We studied *Prkar2a-* haploinsufficient (*Prkar2a*^*+/−*^), *Prkar2b-* haploinsufficient (*Prkar2b*^*+/−*^), *Prkar2a*^*−/−*^ KO, as well as double-heterozygote (*Prkar1a*^*+/−*^*x Prkar2a*^*+/*-^) F1 mice for the development of hematologic malignancies. We found that *Prkar2a* animals developed a spectrum of B cell lineage-derived and histiocytic hematopoietic neoplasms (as well as lung and liver tumors) with an incidence significantly higher than those previously reported for *Prkar1a* KO or *Prkar1a*-haploinsufficient mice.

## Methods

### Animal protocol

Mice deficient for *Prkar1a* and *Prkar2*a alleles that have been described previously [8, 12], were maintained on a mixed genetic background (C57BL/6/129Sv) and were crossed to generate *Prkar2a*^+/−^ and *Prkar2a*^−/−^ mice. We also studied *Prkar2b*^*+/−*^ mice on the same mixed genetic background. All studies were performed under animal protocol 12–033 and were approved by and conducted in accordance with the *Eunice Kennedy Shriver* National Institute for Child Health and Human Development Institutional Animal Care and Use Committee. Mice have been observed between the ages of 5 and 25 months and were necropsied when found to have splenomegaly, lymphadenopathy, hepatomegaly, labored breathing, ruffled fur, or other signs of significant morbidity. At necropsy, harvested tissues were fixed in 10 % neutral buffered formalin for histopathologic study and other samples were frozen for subsequent DNA, RNA and protein studies.

### Flow Cytometry Analysis (FACS)

Single-cell suspensions were stained with mAb conjugated to FITC, PE, PerCP, APC, or biotin (BD Biosciences) assayed on a FACSCalibur (Becton Dickinson). The data were analyzed with FlowJo (TreeStar Inc) or WinMDI (The Scripps Institute) software. The following antibodies were used: B220-PercP (BD553093), IgM-APC (BD550676), CD43-APC (BD560663), CD24 (HSA)-APC (BD562349), Ly-1 (BP-1)-PE (BD496578), CD3e-PE-Cy™7 (BD552774), CD4-PE (BD553730), CD8-APC (BD553035).

### Immunohistochemistry

Formalin fixed, paraffin embedded sections were stained with hematoxylin and eosin (H&E) and antibodies (B220 antibody (dilution 1:50), BD Pharmingen, 550286 and Mac-2 (Galectin-3) antibody (dilution 1:500), Biolegend, 810801). Histologic diagnoses of hematopoietic neoplasms were made using established criteria [13–16].

### Southern blot hybridization

DNA prepared from frozen samples of spleen obtained at necropsy was processed for Southern blot hybridization using established procedures and hybridized with an IgH J_H_ probe [15, 17–19].

## Results

### Tumor development in *Prkar1a*-, *Prkar2a*- and *Prkar2b*-deficient mice

*Prkar1a*^*+/−*^*, Prkar2a*^*+/−*^*, Prkar2a*^*−/−*^, *Prkar2b*^*+/−*^ and wild type (WT) mice were necropsied when they developed splenomegaly, lymphadenopathy (at any age) or when moribund between 5 and 25 months of age. Tissues from a total of 31 mice were studied.

**Fig. 1:**
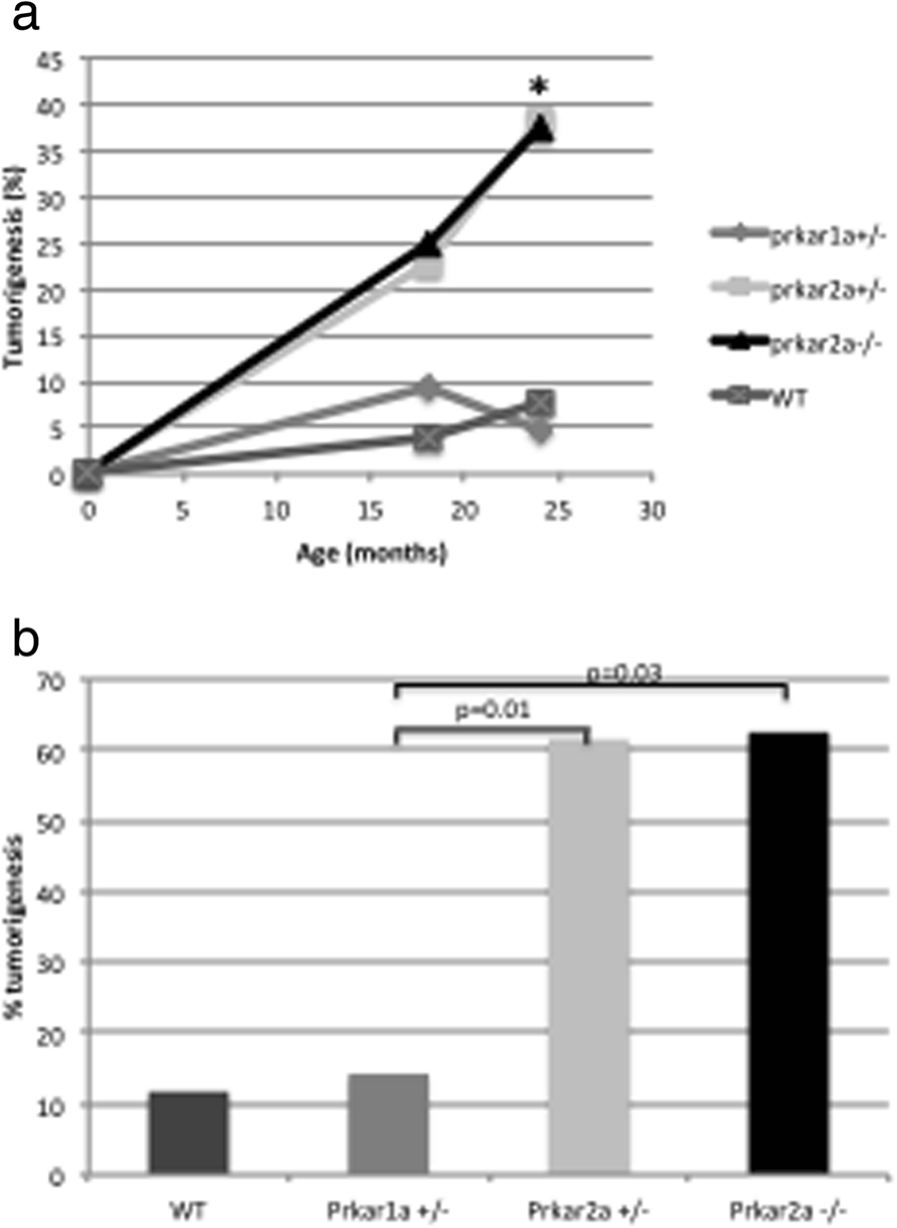
Incidence and time course of tumor development in *Prkar1a* ^*+/−*^ *(n = 28), Prkar2a* ^*+/−*^ *(n = 21), Prkar2a* ^*−/−*^ (*n* = 5) and wild type (WT) (*n* = 25) mice. All mice were on a mixed (C57BL/6/129Sv) genetic background. **a** Total tumor incidence among mice followed for 25 months; * = *p* < 0.02. **b** Frequency of tumors among mice necropsied at 9 and 25 months of age

*Prkar2a-*deficienct mice developed hematopoietic tumors with higher frequency than *Prkar1a*-deficient and WT mice (Fig. 1). Remarkably, a total of 16 cases (51.6 %), including 11/19 cases from the *Prkar2a*^*+/−*^ mice (58 %), were diagnosed as histiocytic sarcomas (HS) [11] (Table 1). Seven others cases were tumors of B cell lineage origin, including diffuse large B cell lymphomas (DLBCL), a plasmocytoma and marginal zone lymphomas (MZL) (Table 1) [13, 20–22].

**Table 1:** Summary of all tumors developed in the animals during the course of the study

**Mouse ID**	**Sex**	**Age**	**Genotype**	**Diagnosis**	**Additional findings**
3209	male	18 mon	*Prkar2a +/−*	DLBCL and HS rich	
3150	female	18 mon	*Prkar2a +/−*	DLBCL	
3345	male	20 mon	*Prkar2a +/−*	MZL	
3485	male	22 mon	*Prkar2a +/−*	HS	
2378	male	18 mon	*Prkar2a +/−*	HS	Lung adenoma
2379	male	18 mon	*Prkar2a +/−*	HS	
2552	female	25 mon	*Prkar2a +/−*	HS	
3078	female	23 mon	*Prkar2a +/−*	HS	
3305	female	24 mon	*Prkar2a +/−*	Liver, Lung, Pancreas: lymphoid infiltrates	Liver: lymphoplasmatic infiltrates
3210	male	24 mon	*Prkar2a +/−*	HS	
3211	male	24 mon	*Prkar2a +/−*	Liver hemangiosarcoma	
3217	male	16 mon	*Prkar2a +/−*	DLBCL and HS	
2622	male	17 mon	*Prkar2a +/−*	HS	
MS1200161	male	16 mon	*Prkar2a +/−*	HS	
3306	female	19 mon	*Prkar2a +/−*	HS	
2494	female	18 mon	*Prkar2a +/−*	HS	
3794	male	6 mon	*Prkar2a +/−*	Spleen Lymphoid Hyperplasia	
2545	male	20 mon	*Prkar2a +/−*	Plasmacytoma	
3753	female	6 mon	*Prkar2a +/−*	Spleen Lymphoid Hyperplasia	
4071	female	18 mon	*Prkar2a −/−*	HS	
4072	female	18 mon	*Prkar2a −/−*	HS	
2537	female	18 mon	*Prkar2a −/−*	DLBCL and HS rich	
3988	female	15 mon	*Prkar1a +/−; Prkar2a +/−*	Liver - Early adenoma	
2383	female	24 mon	*WT*	HS	
3146	female	18 mon	*WT*	EMZ	
3852	male	17 mon	*WT*	MZL	
3437	male	18 mon	*Prkar2b +/−*	Lymph nodes: plasma cell tumor	
3436	female	18 mon	*Prkar2b +/−*	Lung: adenoma	Spleen plasma cells
3147	female	18 mon	*Prkar2b +/−*	EMZ	
2860	female	25 mon	*Prkar2b +/−*	HS	
1941	female	16 mon	*Prkar2b +/−*	DLBCL	
2204	female	24 mon	*Prkar2b +/−*	HS	
2939	female	24 mon	*Prkar1a +/−*	MZL	Lymph nodes plasmacytosis
3069	male	24 mon	*Prkar1a +/−*	Lung: fibrosarcoma; adenoma	
3087	male	22 mon	*Prkar1a +/−*	HS	
1354	male	23 mon	*Prkar1a +/−*	Early HS	Trachea lymphoplasmacytic

*HS* Histiocytic sarcoma, *DLBCL* Diffuse large B cell lymphoma, *EMZ* Expanded marginal zone, *MZL* Marginal zone lymphoma

**Fig. 2:**
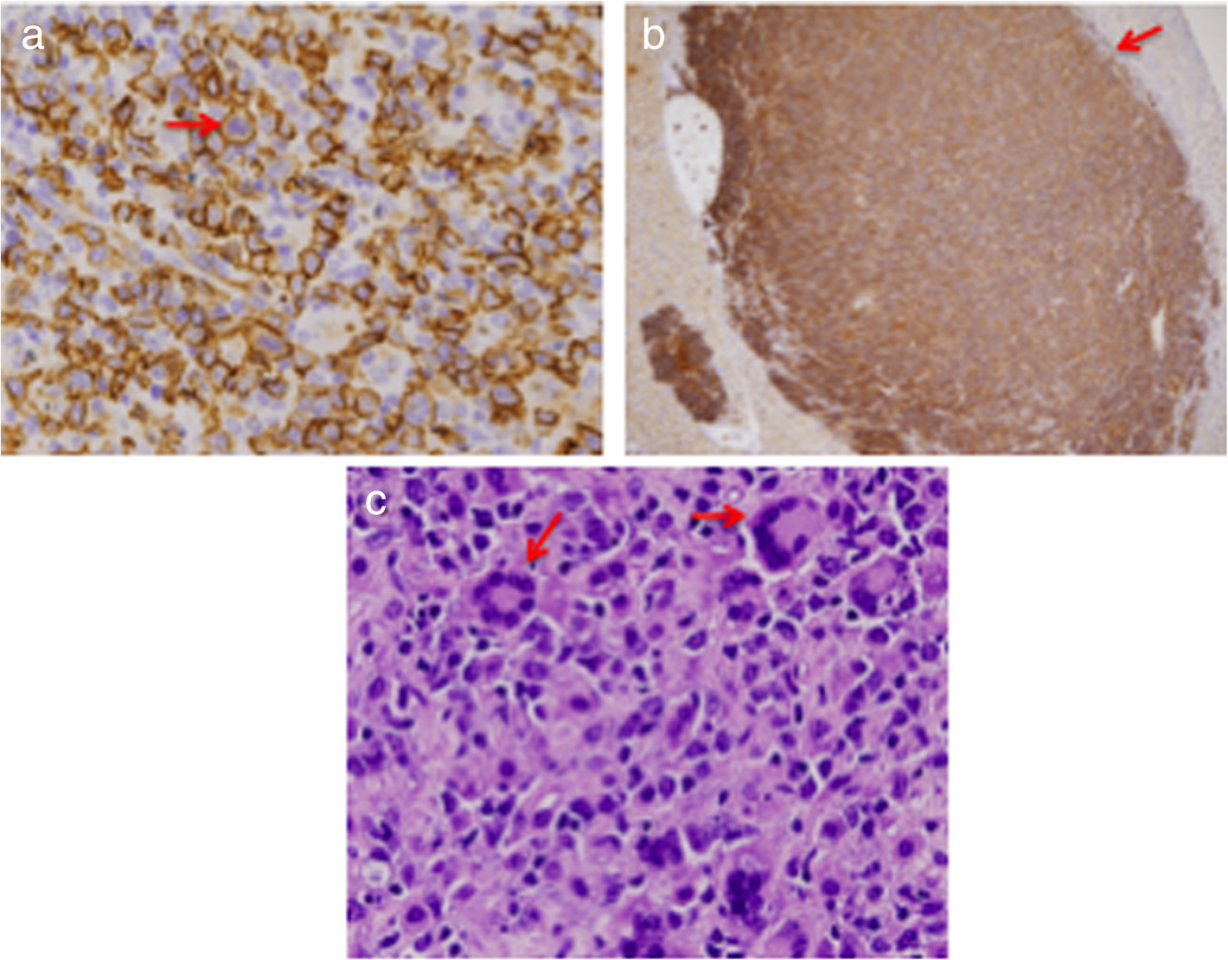
Histopathology and immunohistochemistry of coexisting DLBCL and HS. Sections of spleen (magnification 40x) (**a**) and liver (magnification 4x) (**b**) from the same mouse were stained with antibody to B220 (red arrows). **a** section from another portion of the same spleen stained with H&E reveals features consistent with the diagnosis of HS including many multinucleate giant cells (red arrows) (magnification 40x) (**c**)

Overall, the most frequent neoplasms were hematopoietic in origin and included diffuse large B cell lymphomas (DLBCL) with 3 of 4 of these cases (Table 1, mice 3209, 3217, 2537, 3150) being histiocyte-rich. In one of these cases, the mouse appeared to have a coexisting DLBCL and a HS (Fig. 2, Table 1 mouse #3217). The spleen of this mouse was infiltrated by DLBCL that stained intensely with antibody to the B cell marker, B220 (Fig. 2a) and had metastasized to the liver. The liver mass stained uniformly with B220 (Fig. 2b) but was negative for the macrophage marker, Mac-2 (data not shown). In another part, the same spleen was essentially replaced with a HS associated with the presence of large multinucleate cells (Fig. 2c) that are often seen in mouse as well as human HS [23].

**Fig. 3:**
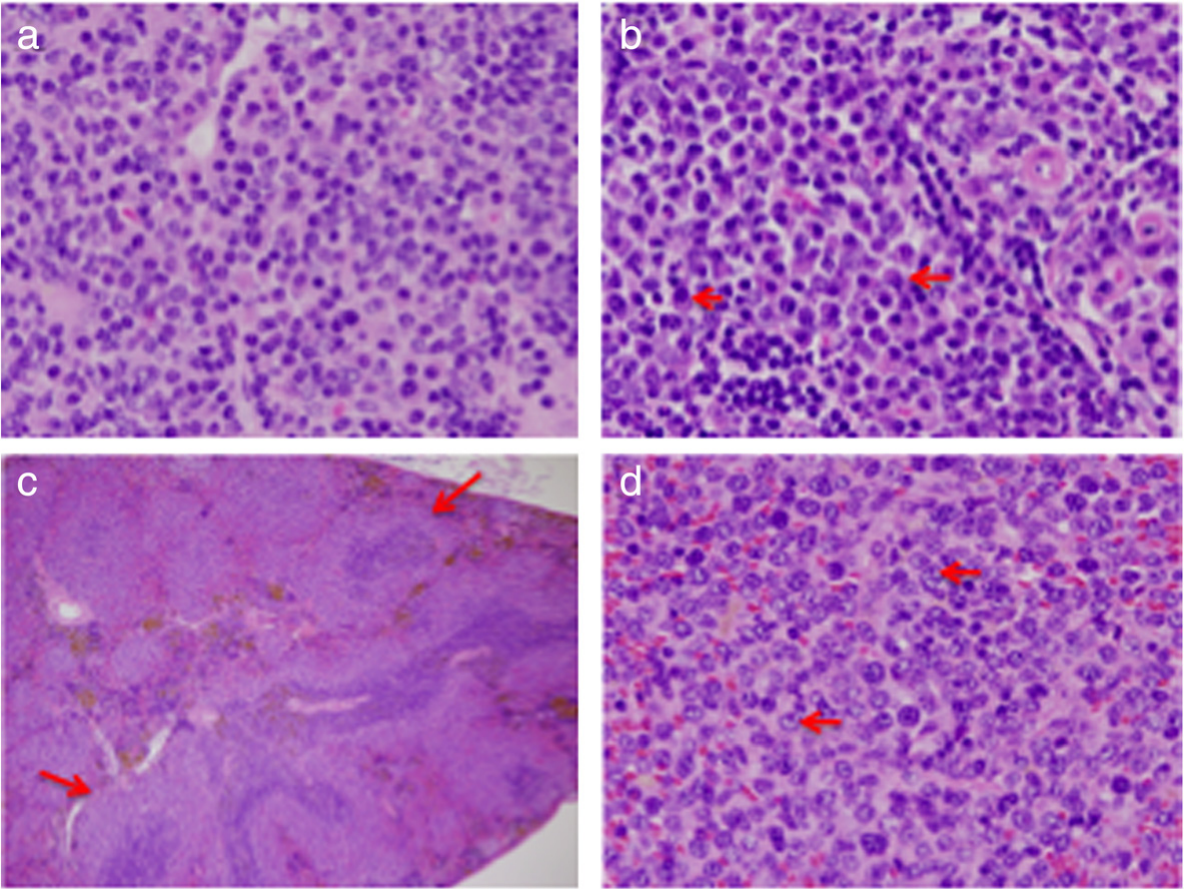
Plasma cell tumors and high-grade splenic marginal zone lymphoma. **a, b** Lymph node sections from two mice (from different litters) stained with H&E (magnification 40x). **a**. Low-grade tumor with mature plasma cells. **b** Higher grade tumor dominated by plasmablasts (red arrows) (**c**) Low power view of a spleen stained with H&E showing small follicles surrounded by multiple layers of pale-staining marginal zone cells (red arrows) (magnification 4x). **d** Cells in the marginal zone have acquired centroblastic cytology (red arrows) (magnification 40x)

Other mice developed malignancies of plasma cells involving the spleen and lymph nodes (Table 1, mice #2545, 3437, 3436, 2939, 3305, 1354). One case (#3437) had sheets of easily recognizable fully mature plasma cells with few mitotic figures (Fig. 3a). A second case (#2545) had numerous plasmablasts with round nuclei containing a large central nucleolus and thick nuclear membranes (Fig. 3b).

We identified a case of high-grade splenic marginal zone lymphoma [24] (Table 1, mouse #3345). At low power, sheets of pale staining cells can be seen, surrounding the follicles and invading both the follicles and the red pulp (Fig. 3c). At high power, the marginal zone is seen to be populated by centroblasts with one or more prominent nucleoli at the nuclear membrane and some immunoblasts with a large magenta bar-shaped nucleolus appended to one side of the nuclear membrane (Fig. 3d).

### DNA studies

**Fig. 4:**
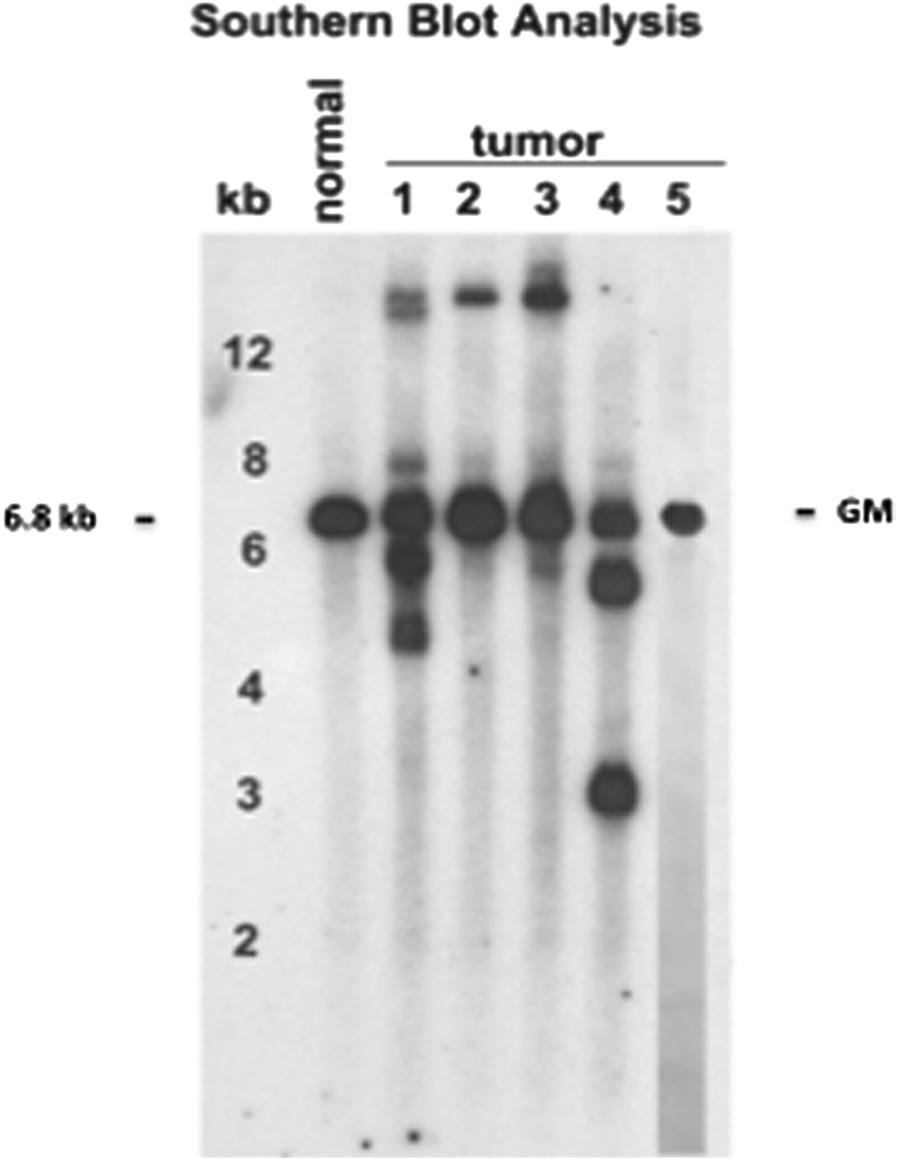
Southern blot hybridization of DNA prepared from 4 DLBCL (Lanes 1–4), a HS (Lane 5) and a normal spleen hybridized with a J_H_ probe. Germline band of 6.8 kb is indicated

To determine if the B cell lineage tumors were clonal, we performed DNA extraction from 4 cases of DLBCL (Table 1, mice # 3217, 3150, 3209, 2537), 1 HS (Table 1, mouse #2622) and a normal spleen and analyzed them by Southern blot hybridization for organization of the IgH locus (Fig. 4). All the DLBCL cases exhibited clonal or oligoclonal rearrangements of IgH while the HS and normal spleen, as expected, had only a 6.8 kb band characteristic of the germline sequence. It is noteworthy that the intensity of the germline band was reduced in only one of the cases of DLBCL indicating that the tumors were not the dominant cell types in the other cases. We concluded from these studies, that the tumors diagnosed histologically as DLBCL were clonal B cell neoplasms.

### Cell sorting

To determine if altered *PRKAR2A* expression might affect early events in hematopoietic differentiation in the BM or the development and distribution of cell subsets in peripheral lymphoid tissues, we performed extensive multiparameter FACS analyses of single cell suspensions prepared from bone marrow (BM), spleen, lymph nodes and thymi from 3–6 month old *Prkar2a*^*+/−*^ and *Prkar2a*^*−/−*^ mice. The analyses were for B cells, T cells and their subsets, macrophages, dendritic cells and granulocytes. We found no significant changes in any cell subset in any tissue from data obtained with WT mice (data not shown). These results indicate that if aberrant *PRKAR2A* expression affected hematopoietic differentiation, the effects only became evident well into adult life.

### Non-hematopoietic tumors

**Fig. 5:**
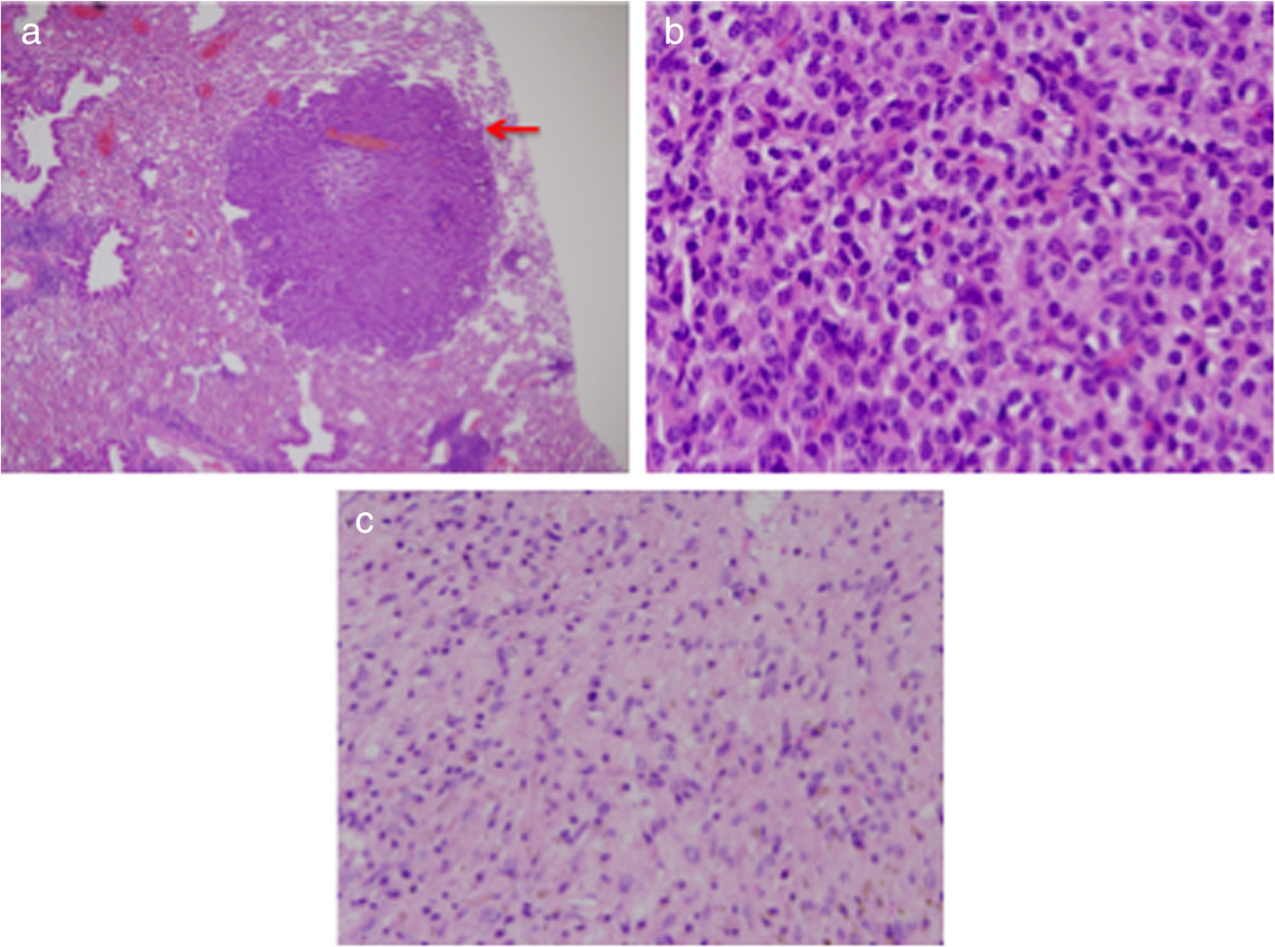
Lung tumors. Pulmonary adenoma (**a**), (magnification 4x) and same pulmonary adenoma at higher magnification (**b**) (magnification 40x). **c** fibrosarcoma (magnification 20x) (**c**)

We identified mice with tumors of the lung (Table 1, mice # 2378, 3436, 3069). These included a pulmonary adenoma (Fig. 5a, b mouse #2378) and a pulmonary fibrosarcoma [21] (Fig. 5c, mouse #3069). The adenoma, which presented as a round mass lesion, was comprised of monomorphic well-differentiated cells with complete loss of normal alveolar architecture. In contrast, the fibrosarcoma presented as a large mass lesion displacing all normal lung elements and was associated with a large necrotic area. It is not known if the lung was where this tumor originated from, but sarcomatous lesions were not identified in other tissues of this mouse.

**Fig. 6:**
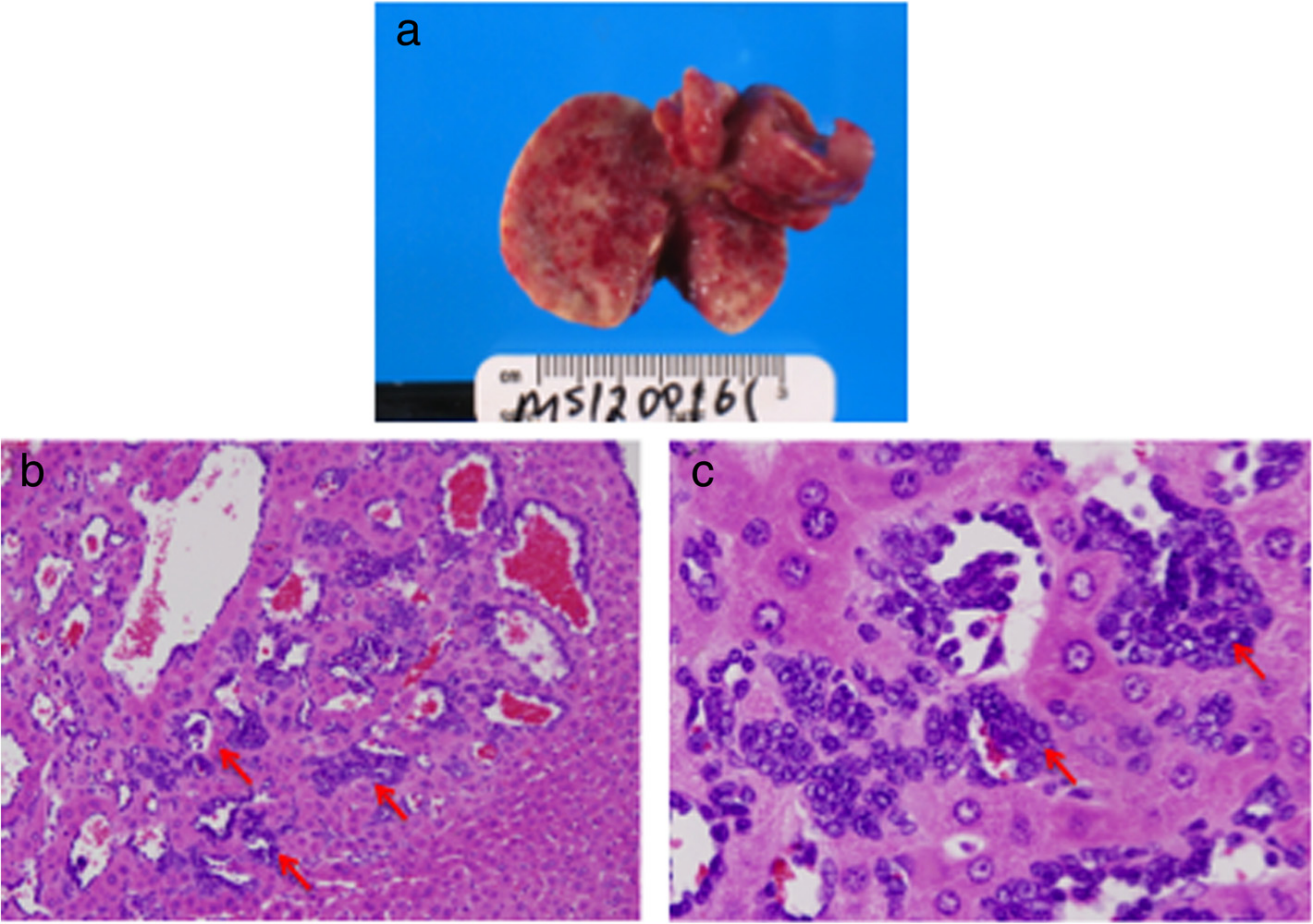
Gross and microscopic presentation of a hepatic hemangiosarcoma. **a** Multiple liver hemangiomas were evident at necropsy. **b, c** Liver sections showing small vascular lesions (*red arrows*) (magnification 10x) (**b**) associated with malignant endothelial cells (*red arrows*) (magnification 40x) (**c**)

One mouse (#3211) developed an uncommon liver vascular lesion readily evident at necropsy (Fig. 6a). Histologic sections of liver revealed features consistent with a diagnosis of malignant hemangiosarcoma but that also resembled *peliosis hepatis*, a non-malignant lesion in which multiple blood filled cavities can be found throughout the liver [25] (Fig. 6b, c).

## Discussion

Our findings in this study identify *Pkrar2a* as a gene whose deficiency predisposes mice to a spectrum of mostly hematopoietic neoplasms. This phenotype has some similarities but also differences from the spectrum of tumors exhibited by humans and mice with RIα defects [6, 9, 26]. The major difference is that *Prkar2a*^*+/−*^ and *Prkar2a*^*−/−*^ mice develop with a high frequency hematopoietic neoplasms, particularly macrophage-derived HS tumors, as well as neoplasms derived from mature cells of the B cell lineage. Lymphomas or HS have not been reported in patients with CNC but were among the tumors found relatively rarely in *Prkar1a*-deficient mice [8, 9]. This suggests that alterations in the RIα and RIIα signaling in different cell types may preferentially predispose them to transformation. It has been shown, for example, that *PRKAR1A* inactivation promotes the proliferation of human B cells in association with reduced apoptosis [27], both of which may enhance B cell sensitivity to malignant transformation.

HS is an uncommon lesion in humans and mice and the etiology in either species remains unknown [16, 23]. Studies of *Prkar2a*-deficient mice revealed that many animals without clear histologic evidence of HS exhibited histiocytic proliferation in association with DLBCL, a condition we refer to as histiocyte-rich DLBCL [13, 28] or, isolated, in the absence of other tumors. It is not clear if such proliferation might be the precursor to tumor development. However, such progression appears possible since in a few animals distinct areas of the spleen were occupied by HS with other areas showing classic features of histiocyte-rich DLBCL. Rarely, HS have been found to harbor IgH or TCR rearrangements, likely as the result of trans-differentiation [29], but this was not found to be the case with the single HS tested here for IgH organization.

It remains to be determined how dysregulation of *PRKAR2A* expression contributes to transformation of these cells. There is a report of a single in-frame expressed fusion gene resulting from a chromosomal translocation in breast cancer between *PRKAR2A* and *SLC26A6*, which encodes an anion transporter [30], but the oncogenetic significance of this event has not been determined. A comprehensive review of the cancer genome atlas (TCGA) did not reveal *PRKAR2A* mutations in any human tumors but several studies show copy number changes of *PRKAR2A*’s chromosomal locus at 3p21 which, however, is gene-rich and is frequently lost in a variety of neoplasms [31–35].

In conclusion, the present study supports the notion that alterations in PKA signaling in a variety of cell types, including those of the hematologic lineage, may lead to neoplastic transformation. *Prkar2a-* deficiency, in particular, predisposes mice to a spectrum of mostly hematopoietic neoplasms, which may lead to better understanding of the molecular pathology of these lesions in both mice and humans.

## Abbreviations

PKA: Protein Kinase A
WT: Wild Type
DLBCL: Diffuse Large B Cell Lymphoma
CNC: Carney Complex
HS: Histiocytic Sarcoma
KO: Knockout
H&E: Hematoxylin and Eosin
MZL: Marginal Zone Lymphoma
HR: Histiocytic Rich
FACS: Fluorescence Activated Cell Sorting
BM: Bone Marrow
TCR: T Cell Receptor
